# Associations between infant social withdrawal and disorganized attachment

**DOI:** 10.1007/s00787-026-03057-9

**Published:** 2026-05-11

**Authors:** Anne Christine Stuart, Katrine Røhder, Sophie Reijman, Ida Egmose, Johanne Smith-Nielsen, Eva Back Madsen, Katrine Isabella Wendelboe, Mette Skovgaard Vaever

**Affiliations:** 1 Centre of Excellence in Early Intervention and Family Studies, Department of Psychology, University of Copenhagen, Øster Farimagsgade 2A, Copenhagen, 1353 Denmark; 2 Department of Clinical Child and Family Studies, Vrije Universiteit Amsterdam, Amsterdam, The Netherlands; 3 National Institute of Public Health, University of Southern Denmark, Copenhagen, Denmark

**Keywords:** Disorganized attachment, Infant social withdrawal, Maternal postpartum depression

## Abstract

Disorganized attachment is an important risk factor for later child development but can only be reliable identified from 11 months of age. To prevent adverse child development, it is essential to identify factors associated with disorganized attachment that can be reliably assessed earlier in development. The present study investigates two potential risk factors, infant social withdrawal and maternal postpartum depression symptom severity, in a sample of 203 Danish mother-infant dyads. Infant social withdrawal (assessed using the Alarm Distress Baby Scale) and postpartum depression symptom severity (assessed using the Edinburgh Postnatal Depression Scale) were measured when the infant was between 2 and 11 months old. Attachment quality was assessed using the Strange Situation Procedure when the infant was between 11 and 20 months. Only infant social withdrawal was significantly associated with disorganized attachment, with indications of this being due to the infant’s ability to engage in interactions. These findings highlight the importance of investigating infant behavior as a potential factor associated with later disorganized attachment.

**Supplementary Information:**

The online version contains supplementary material available at https://doi.org/10.1007/s00787-026-03057-9.

## Introduction

The quality of infant-parent attachment relationship is central to children’s socio-emotional development and well-being [1, 2]. Disorganized attachment is characterized by infants’ apparent lack of a coherent and organized emotion regulation strategy to seek comfort from their caregiver when distressed [3]. It has been linked to later psychopathology, impaired social competence, and poorer school adaptation [1–3]. Although other methods exist for assessing child attachment, the Strange Situation Procedure (SSP; [4]) is considered the most reliable method to assess attachment quality [5], but can only be reliably used from 11 months of age [4]. For prevention, it is essential to identify early risk factors for disorganized attachment that can be reliably assessed during the first year of life. As part of a larger randomized controlled trial (RCT), the present study investigates two such early factors: maternal postpartum depressive symptoms and infant social withdrawal.

The attachment and exploratory systems are interlinked, mutually inhibiting behavioral systems [6]. Secure attachment is characterized by the caregiver’s ability to serve as a secure base for exploration when the child is not distressed and as a safe haven to provide comfort and emotion regulation during distressing situations [6]. A balance between the two systems is thus characteristic of secure attachment (B). When the caregiver is unable to fulfill one/both of these roles, the risk of child insecure attachment (avoidant and resistant) increases: Children with avoidant attachment (A) will explore but display little secure base behavior, and children with resistant attachment (C) will show resistant and passive behavior to elicit responses from the caregiver at the expense of their exploration. Although suggestive of an imbalance between the attachment and exploratory systems, the two insecure attachment strategies are considered organized emotion regulation strategies functional in the context of the relationship. Disorganized attachment (D), on the other hand, was developed as an “umbrella” category comprising a wide variety of inexplicable and/or contradictory attachment behaviors [7]. During the SSP, infants typically show an identifiable pattern of organized attachment (A, B, or C) which can be disrupted by disorganized behaviors. If the behaviors are repeated or happen at significant moments, the child may be assigned a primary classification of disorganized attachment. Although most research has focused on parental behaviors as predictors of disorganized attachment, meta-analytic evidence suggests that maternal insensitivity (*r* =.19) and atypical parental behavior (*r* =.34) account for only part of the variance in disorganized attachment [5, 8], highlighting the need to examine other contributing factors, including the infant’s behavior [8, 9].

Sustained infant social withdrawal is a sign of distress and reduced engagement in social interactions with both caregivers and strangers [10]. It is typically assessed using the Alarm Distress Baby Scale (ADBB; [10]) during interaction with a trained observer (e.g., clinician, health visitor), capturing withdrawal in general social interactions [11]. Social withdrawal is not a diagnosis but is considered an alarm signal for congenital conditions and/or inadequate caregiving [12]. Although assessed with a stranger, infant social withdrawal is associated with lower maternal sensitivity and more intrusiveness [13, 14], suggesting links to broader relational functioning. Social withdrawal has also been linked to a range of developmental difficulties, including non-organic failure to thrive, developmental disorders, behavioral problems, and infant psychopathology [10, 13, 15], cognitive, motor, and language development as well as relational and behavioral disorders [16–18]. It is thus considered an important early risk marker of child development and psychological difficulties. Only two studies, to our knowledge, have examined its relationship with attachment quality using the SSP. The first study found no significant correlations, likely due to its small sample size (*N* = 23) [19]. The second study had a larger sample (*N* = 94) and found an increased risk of insecure attachment among withdrawn infants [20]. Interestingly, neither included infants classified as disorganized.

Another potential early factor is postpartum depression; a common diagnosis, affecting 12–17% of otherwise healthy women [21] with the potential to negatively impact parent-child interaction [22]. Depressive symptoms have been associated with more severe infant social withdrawal [23], potentially through transactional processes: Initial infant withdrawal may lead to the caregiver feeling rejected (which is linked to maternal depression), prompting more anxious and intrusive caregiving, which the infant then experiences as threatening or alarming, resulting in more infant social withdrawal [13]. A recent study found that atypical maternal behavior mediates the effect of maternal depression on social withdrawal in six-months-old infants [24]. Meta-analytic evidence indicates that parental psychopathology, including depression, increases the likelihood of disorganized attachment [5], particularly when symptoms are chronic or severe [25, 26].

The present study examined whether maternal postpartum depression symptom severity (assessed with the Edinburgh Postnatal Depression Scale; EPDS [27]) and infant social withdrawal (assessed with the ADBB [10]) were associated with disorganized attachment (assessed with the SSP). As previous research has found a link between EPDS and ADBB (e.g. [23]),, we also explored if they had a cumulative effect. If associations were identified, we examined whether the predictors were specific to disorganized attachment or extended to the child’s secondary attachment classification (i.e., the identifiable organized attachment pattern disrupted by disorganized behaviors). Finally, to inform clinical practice, we explored whether specific ADBB and/or EPDS items and total scores best distinguished between disorganized and organized attachment.

## Methods

### Participants and procedure

The present study was part of the Copenhagen Infant Mental Health Project (CIMHP; [28]) and was conducted in accordance with the ethical standards for research and approved by the university’s ethical review board (approval number: 2015-10).

Families were recruited between July 2015 and July 2019 by the health visitor who routinely visits the family in the first year postpartum as part of the national health care services in Denmark. Mothers scoring above cut-off on EPDS (≥ 10) and/or infants scoring above cut-off on ADBB (≥ 5) were referred to the project. Soon after referral, a project psychologist conducted a home visit to assess eligibility by confirming maternal depression and/or sustained infant social withdrawal, defined as continued scoring above cut-off. Referrals could occur between 2 and 11 months postpartum, although most families were included when infants were 2–3 months old (M = 3.31, SD = 2.19, 80.8% of the sample). Exclusion criteria included maternal age under 18 years; insufficient Danish language proficiency; bipolar disorder/psychotic disorder, known severe intellectual impairment, or alcohol/substance abuse; suicide attempt during pregnancy or postpartum; infant gestational age below 28 weeks; and severe infant medical conditions.

Informed written consent was obtained, and data (i.e., EPDS and ADBB assessment for the present study) was collected. The families were then randomized within an RCT to either the Circle of Security – Parenting (COSP™) program [29] or care-as-usual. At follow-up, when infants were at least 11 months (*M* = 13.83, *SD* = 1.59, range 11–20 months), families attended a laboratory assessment, including the SSP to assess attachment quality (see Table S1 for procedural details).

Because COSP™ aims to promote secure attachment, it could potentially confound the present analyses. However, the intervention showed no effects on attachment or other related parenting outcomes, e.g., sensitivity and mentalizing, in this sample [30], consistent with a systematic review reporting no conclusive evidence of COSP™ affecting child-parent attachment [31].

### Measures

#### Infant social withdrawal

The ADBB is an observer-rated instrument assessing infant social withdrawal during interaction with a stranger. It comprises eight items (facial expressions, eye contact, general level of activity, self-stimulating gestures, vocalizations, response to stimulation, relationship with the observer, and capacity to attract and maintain the observer’s attention) that are rated on a Likert scale from 0 to 4. Items are summed to a total score, with higher scores reflecting more social withdrawal. A cut-off score of 5 has generally been recommended [12].

During the home visit, the infant interacted with the project psychologist (i.e., the stranger) who conducted a live ADBB rating based on this interaction. The present study uses the total score from this assessment. To ensure reliability, all home visits were video recorded, and 20% of the sample was randomly selected for reliability coding by a trained and certified ADBB rater (6th author). Interrater reliability was assessed using intraclass correlation coefficients (ICC; two-way random effects consistency single rater model [32]) and was good (ICC = 0.79, 95% CI [0.72; 0.84]; Cohen’s κ = 0.66). The same assessor conducted both the EPDS and ADBB during the home visit, although the order varied to accommodate the family. Thus, the live ratings were not blinded. In contrast, the reliability coder was blind.

#### Maternal postpartum depressive symptoms

The EPDS was used to assess symptoms of postpartum depression occurring in the past seven days for the mother. The self-report questionnaire consists of 10 items, relating to mood and feelings, and rated on a 4-point Likert scale. Total scores range from 0 to 30, with higher scores indicating more severe symptoms. In the analyses, we used the total score from the visit of the project psychologist.

#### Child-mother attachment quality

The SSP is a validated experiment for evaluating the quality of attachment relationships when the infant is 11–24 months old. In a maximum 20-minute laboratory observational procedure, the dyads are observed for eight brief episodes with the mother and infant being introduced to an unfamiliar room, a stranger enters the room, and the mother leaves twice. During the child’s reunions with the mother, their attachment behaviors are observed and assigned a classification (A, B, or C). Disorganized behavior is given a score, ranging from 1 to 9. If a child scores above 5, they are given a disorganized classification. Attachment quality was coded by the third and fourth authors. 44 randomly selected videos were reliability coded. Interrater reliability was substantial for the three-way ABC classification (Cohen’s κ = 0.74) as well as the four-way ABCD classification (Cohen’s κ = 0.67).

### Statistical analyses

All analyses were conducted in R v. 4.3.2. The *p*-values were two-tailed and evaluated at a significance level of 0.05. Using binary logistic regressions, we assessed whether potential covariates were associated with disorganized attachment. To test our hypothesis, we estimated two binary logistic regression models examining associations between ADBB and EPDS scores and disorganized attachment, using (1) secure attachment and (2) organized attachment as reference groups. An interaction term was included to explore the cumulative effect of infant social withdrawal and maternal postpartum depression symptoms.

The following analyses were exploratory. We examined associations with children’s secondary attachment classification. For significant total scores, we conducted item-level analyses by entering all items simultaneously into a binary logistic regression model to assess their unique contributions. Finally, if either ADBB and/or EPDS proved to be significant, we explored what total score best distinguished between organized and disorganized attachment, using receiver operating characteristics (ROC) analyses (ROCit v. 2.1.1). Discriminative ability was assessed using the area under the curve (AUC), with values above 0.5 indicating performance better than chance [33]. Sensitivity (i.e., correctly identifying those children with disorganized attachment) and specificity (i.e., correctly identifying those children with organized attachment) were calculated across all possible scores, and the Youden-index was used to identify the optimal threshold [34].

## Results

Sample characteristics and descriptive statistics are presented in Table 1. The sample was resourceful, with most mothers being in a relationship with the child’s father, highly educated, and employed. Child sex was associated with disorganized attachment (*p* =.036), with boys having higher odds. Neither RCT group (*p* =.164) nor other background variables (all *p*s ≥ 0.095) were associated with attachment. Thus, only child sex was included as a covariate in subsequent analyses. As reported in the RCT outcome study [30], attrition was related to RCT group and family functioning, but not to maternal depression or infant social withdrawal.

**Table 1:** Sample characteristics and descriptive statistics (*N* = 203)

RCT group, *n* (%)		
Care-as-usual	56	(27.6)
Circle of Security - Parenting	147	(72.4)
Risk inclusion		
ADBB	12	(5.9)
EPDS	177	(87.2)
ADBB and EPDS	14	(6.9)
Maternal relationship status, *n* (%)		
In a relationship	179	(88.2)
Single	12	(5.9)
Maternal employment status, *n* (%)		
Employed	138	(68.0)
Unemployed	65	(32.0)
Maternal educational level, *n* (%)		
9–12 years (ISCED level 3)	20	(9.9)
15 years (ISCED levels 4, 5, 6)	96	(47.3)
17 years (ISCED levels 7, 8)	78	(38.4)
Maternal age at birth, years, *M* (*SD*)	32.25	(4.68)
Infant age at baseline, months, *M* (*SD*)	3.31	(2.19)
Infant sex, *n* (%)		
Boy	105	(51.7)
Girl	98	(48.3)
Child-mother attachment quality		
Avoidant (A)	6	(3.0)
Secure (B)	126	(62.1)
Resistant (C)	26	(12.8)
Disorganized (D)	45	(22.2)
Maternal EPDS score, *M* (*SD*)	14.83	(4.57)
Infant ADBB score, *M* (*SD*)	1.85	(2.30)

Note. *EPDS* Edinburgh Postnatal Depression Scale,* ADBB* Alarm Distress Baby Scale,* ISCED* International Standard Classification of Education

Results from the binary logistic regression analyses are presented in Table 2. EPDS scores were not significantly associated with disorganized attachment, but ADBB was, indicating that higher ADBB scores were associated with odds increasing by 1.21 for disorganized compared to secure attachment. This association remained significant when comparing disorganized to organized attachment. The interaction between EPDS and ADBB was non-significant for both secure vs. disorganized (OR = 0.98, *p* =.216, 95% CI [0.95; 1.01]) and organized vs. disorganized attachment (OR = 0.98, *p* =.146, 95% CI [0.95; 1.01]). Including RCT group as a covariate did not alter the results, and ADBB remained significantly associated with disorganized attachment (*p* =.033).

**Table 2:** Results of logistic regression models with ADBB and EPDS as predictors controlled for the influence of infant sex

	Secure vs. disorganized			Organized vs. disorganized		
	OR	*p*	95% CI	OR	*p*	95% CI
EPDS	1.02	0.634	[0.94; 1.10]	1.01	0.774	[0.94; 1.09]
ADBB	1.21	0.013	[1.04; 1.41]	1.17	0.029	[1.02; 1.34]

Note. *EPDS* Edinburgh Postnatal Depression Scale, *ADBB* Alarm Distress Baby Scale. Secure and organized were the reference groups in the respective models

Four children had avoidant, 20 secure, and 18 resistant secondary attachment classifications. Three children could not be given a secondary attachment classification and were excluded. Due to the small number of avoidant cases, these were not included in the analysis. ADBB scores were not significantly associated with secondary attachment classification (OR = 0.90, *p* =.423, 95% CI [0.67; 1.19]).

As only the total score of the ADBB was associated with disorganized attachment, subsequent exploratory analyses focused on ADBB.

In an explorative item-level logistic regression model (Table 3), item 6 was excluded due to all children scoring 0. Only item 7 (relationship with the observer) was significant, indicating it may drive the association between ADBB and disorganized attachment. However, this did not remain significant after correction for multiple testing with the Benjamini-Hochberg procedure (*p* =.012, adjusted α = 0.0071).

**Table 3:** Explorative analyses of ADBB items’ association with disorganized compared to organized attachment

	OR	*p*	95% CI
Item 1: Facial expression	0.58	0.232	[0.24; 1.42]
Item 2: Eye contact	0.95	0.892	[0.44; 2.05]
Item 3: General level of activity	0.49	0.355	[0.11; 2.23]
Item 4: Self-stimulating gestures	0.43	0.270	[0.10; 1.92]
Item 5: Vocalizations	0.95	0.980	[0.51; 1.88]
Item 6: Response to stimulation	-	-	-
Item 7: Relation	2.97	0.012	[1.27; 6.94]
Item 8: Attraction	1.08	0.841	[0.50; 2.32]

Note. *ADBB* Alarm Distress Baby Scale

Finally, ROC analyses (Table 4) explored the ability of the ADBB to distinguish between organized and disorganized attachment. The AUC was 0.58 (95% CI [0.51; 0.64]), indicating performance slightly above chance. The Youden-index suggested an optimal score of 3, balancing between sensitivity and specificity. Of the 45 children with disorganized attachment, 19 (42%) scored above 3 on the ADBB.

**Table 4:** Results from the ROC analysis

	Sensitivity	Specificity
ADBB scores		
5+	21.74	87.34
4+	26.09	82.28
3+	43.48	71.52
2+	52.17	60.13
1+	63.04	44.94

## Discussion

The aim of the present study was to examine whether maternal postpartum depression severity and infant social withdrawal, assessed when infants were 2–11 months old, were associated with disorganized attachment at 11–20 months of age.

We did not find postpartum depression severity to be associated with disorganized attachment, which was unexpected. A recent meta-analysis [5] reported a higher prevalence of disorganized attachment in clinical samples (23%), comparable to the prevalence observed in our sample (22.2%). However, the authors emphasized the importance of considering factors such as chronicity, timing, severity, and their impact on parental behavior. Although most mothers met the diagnostic criteria for depression at baseline, this was not necessarily the case at follow-up when attachment was assessed. A systematic review suggested that only depression within the first six months has an impact on child attachment [26], however, this is unlikely to fully account for our null-finding, as most mothers were assessed within this period (85.3%). A more plausible explanation may be limited variability in depressive symptoms, as 87.2% of participants were recruited based on elevated EPDS scores. Additionally, the relatively well-resourced nature of the sample may have buffered potential effects of maternal depression, consistent with a previous study in a similar sample [35]. Future studies should further investigate how characteristics of postpartum depression relate to disorganized attachment.

We found that infant social withdrawal was significantly associated with disorganized attachment, with each one-point increase on the ADBB scale raising the odds of disorganized attachment by 1.21. There was no significant association with secondary attachment classification, although only tested in a small group of infants, possibly limiting our statistical power to detect significant relationships. These findings suggest that social withdrawal may be specifically related to the disorganized attachment classification. To our knowledge, this is the first study to empirically investigate this association. This finding may inform theoretical accounts of disorganized attachment. In his unpublished works, Bowlby described three (not mutually exclusive) potential pathways to behaviors that were later operationalized into disorganized attachment: threat conflict, safe haven ambiguity, and activation without assurance [36]. *Threat conflict*, the most widely recognized pathway, suggests that the infant simultaneously perceives the caregiver as a source of safety and threat, disrupting the attachment system. This is reflected in Main and Solomon’s [7] coding system and supported by empirical findings linking frightened-frightening parental behavior to disorganized attachment [8]. *Safe haven ambiguity* occurs when caregivers send highly ambiguous, but not threatening, signals about being a safe haven in situations of distress, potentially leading to infant dysregulation. *Activation without assurance* refers to prolonged caregiver separations, such as placement in foster care or hospitalization [7]. Given that all infants in our study lived with their caregivers, the first two developmental pathways seem particularly relevant in explaining the observed association. Although not assessed in our study, atypical frightened-frightening parental behavior may underlie both behaviors [8, 24]. However, studies also indicate that frightening-frightened behavior is only associated with disorganized attachment in infants with insecure secondary attachment classifications [37, 38], which comprised only 40% of our infants with a disorganized attachment classification in our sample.

An alternative explanation could be that repeated experiences of safe haven ambiguity contribute to a generalized pattern of dysregulation. Caregivers who display muted affect (e.g., in the context of severe depression) or strongly mismatched reactions to the infant’s cues may limit opportunities for co-regulation, leading infants to adopt withdrawal as a regulation strategy. This interpretation is supported by evidence linking maternal atypical behavior to infants’ stress regulation during the SSP [39]. Similarly, social withdrawal has been conceptualized as a sign of dysregulated parent-infant synchrony, as demonstrated in the Still Face Paradigm and experimental designs on desynchronization between mother and infants – both designed to mirror the affective miscommunication seen in mothers with depression [12, 40, 41]. Disruptions in co-regulation and affective communication may thus over time contribute to the development of disorganized attachment. Importantly, the ADBB assesses infant behavior in interaction with a stranger rather than the caregiver. Reduced engagement (item 7) may therefore reflect alternative explanations, including temperamental characteristics such as shyness. However, prior research suggests that this item reflects interpersonal functioning rather than temperament [11], and social withdrawal has not been consistently associated with temperament [14]. This supports the interpretation of withdrawal as reflecting broader interpersonal or regulatory functioning that may generalize across contexts. Consistent with this, our exploratory analysis suggested that item 7 was associated with disorganized attachment, although this did not remain significant after correction for multiple testing. Our exploratory finding may indicate that the infant in new encounters with strangers lacks skills to engage in and elaborate on the interaction and instead chooses a passive or withdrawing coping strategy, potentially mirroring past experiences of affective miscommunication within the dyad with their depressed mother. In support of this pathway, we would have expected to find a cumulative effect of postpartum depression and social withdrawal, which was not significant. However, it is highly likely that our study was underpowered to detect such an interaction effect, and future longitudinal studies should investigate the potential developmental pathways between infant social withdrawal and disorganized attachment. It is important to note that this pathway explanation is only hypothetical, as we have not tested the possible mechanisms linking social withdrawal and disorganized attachment. Future studies should both replicate our findings as well as investigate empirical evidence for these pathways.

Finally, it is also important to consider whether children may have congenital characteristics that underlie the found association between social withdrawal and disorganized attachment. Limited emotion regulation in newborns was associated with disorganized attachment in a combined longitudinal sample [42] and in a partial replication, exploratory research found that limited emotion regulation was not associated with disorganized attachment overall, only with non-fearful forms of disorganized attachment, i.e., disorganized behaviors in which the infant’s inferred fear or alarm is not overtly displayed [43]. As far as we are aware, no research has examined correlations between emotion regulation and ADBB, but infants with a “withdrawn” profile based on behavioral and physiological markers showed limited emotion regulation in one study [20]. Another potential avenue for further exploration is whether some of the children who exhibit social withdrawal and disorganized attachment will later be diagnosed with a neurodevelopmental disorder. Previous studies have shown that infant social withdrawal is an early marker of autism spectrum disorder [44, 45], which is itself associated with increased rates of disorganized attachment [46]. Thus, shared underlying developmental vulnerabilities may partly explain the association observed in the present study.

Clinical practitioners need instruments that feasibly and reliably identify early developmental risks to enable timely interventions that support families. Social withdrawal can be assessed reliably by pediatricians and health visitors as early as two months using the ADBB (e.g. [12, 47]),. The present study supports its utility as an early marker associated with later attachment difficulties. Exploratory analyses suggested that a lower cut-off score (3 rather than 5) may better distinguish between disorganized and organized attachment. This is in line with a previous Norwegian study also suggesting a cut-off of 3 [48]. However, this was not a formal validation study of the cut-off score on the ADBB, and disorganized attachment is not a gold standard for withdrawal. This is also evident from the results, as the sensitivity and specificity indicated that we would still miss over half of the children classified with disorganized attachment, as well as misidentify around a quarter of the children without disorganized attachment. Nonetheless, the AUC indicated that the ADBB was better than chance at identifying children with disorganized attachment, and these results highlight the need for further validating the ADBB cut-off in a Nordic context as well as cross-validating our findings regarding disorganized attachment.

The present study has some limitations that should be considered. First, our sample was at-risk. Given that disorganized attachment is more prevalent in clinical populations [5] and that infant social withdrawal has been associated with postpartum depression [23], the association we found may be stronger than what would be observed in a typical community sample. Second, the sample size was also relatively small, possibly affecting the generalizability of the results as well as the statistical power. Third, some mothers received a parenting intervention (COSP™). As the intervention group was not significantly associated with attachment quality and no interventions effects were found in our primary outcome study [30] nor in a systematic review [31], we felt justified in pooling the groups for the present study. That said, if COSP™ had influenced attachment in this sample, it would most plausibly have attenuated the association between early social withdrawal and later disorganized attachment, as an effective intervention would be expected to shift some high-risk dyads toward more secure attachment classifications. From this perspective, the observed association between ADBB scores and disorganized attachment, despite the intervention, may be considered conservative. However, this reasoning is speculative, and in the absence of baseline attachment assessments, we cannot rule out more complex intervention effects, including differential effects across levels of infant withdrawal. The findings should therefore be interpreted with caution and require replication in purely observational longitudinal designs.

In conclusion, the present study found that early infant social withdrawal was associated with later disorganized attachment. Although a theoretical link has been proposed, this is the first study to provide empirical support for this relationship. Previous research on disorganized attachment has primarily focused on caregiving behaviors, has rarely operationalized the parent-child relationship as a process of co-regulation, and has not included infant behavior as potential predictors. Our findings suggest that infant behavior, specifically sustained social withdrawal may be an important factor in the development of disorganized attachment, and future research should continue to explore how the two constructs are related.

## Supplementary Information

Below is the link to the electronic supplementary material.

Supplementary Material 1 (DOCX 1.67 MB)

## Data Availability

The data that support the findings of the study are available from the authors upon reasonable request.
