# Supplementary material for: Associations between infant social withdrawal and disorganized attachment
Source: Eur Child Adolesc Psychiatry. 2026 May 11;35(9):2779–86. doi: 10.1007/s00787-026-03057-9 (PMC13695589; doi:10.1007/s00787-026-03057-9)
Supplement: Supplementary file 1 [file 787_2026_3057_MOESM1_ESM.docx]

| **Table S1.** Child's age at baseline and calculated optimal age at follow-up assessment | | | |
| --- | --- | --- | --- |
| Child's age (months) at baseline visit | Waiting time (months) between baseline and follow-up assessment | Optimal age (months) at follow-up assessment | Child's age (months) when family is contacted by the research team regarding follow-up assessment |
| 2 | 10 | 12 | 11 |
| 3 | 9 | 12 | 11 |
| 4 | 9 | 13 | 12 |
| 5 | 8 | 13 | 12 |
| 6 | 8 | 14 | 13 |
| 7 | 7 | 14 | 13 |
| 8 | 7 | 15 | 14 |
| 9 | 6 | 15 | 14 |
| 10 | 5 | 15 | 14 |
